# Initiating communities of practice for teaching and education scholarship in hospital settings: a multi-site case study

**DOI:** 10.15694/mep.2018.0000127.1

**Published:** 2018-06-11

**Authors:** Daniel Miller, Debbie Kwan, Stella Ng, Farah Friesen, Mandy Lowe, Jerry Maniate, Lakshmi Matmari, Latika Nirula, Denyse Richardson

**Affiliations:** 1 Centre for Faculty Development, University of Toronto, Toronto, Ontario, Canada; 2 Department of Occupational Science and Occupational Therapy, University of Toronto, Toronto, Ontario, Canada; 3 Faculty of Medicine, University of Ottawa, Ottawa, Ontario, Canada; 4 Toronto Western Hospital, University Health Network, Toronto, Ontario, Canada; 5 Department of Psychiatry, University of Toronto, Toronto, Ontario, Canada; 6 Department of Medicine, University of Toronto, Toronto, Ontario, Canada

**Keywords:** faculty development, education scholarship, community of practice, qualitative research

## Abstract

This article was migrated. The article was marked as recommended.

**Background and Rationale:** Numerous calls have been made for faculty development programming to better address faculty members’ ongoing needs, to situate training strategies within the workplace and to utilize social learning perspectives, communities of practice in particular. Reviews have pointed to a paucity of published qualitative research on faculty development communities of practice and, more generally, on the processes of change and the organizational contexts in which interventions are implemented.

**Intervention:** An initiative was started to instigate education scholarship communities of practice in three highly distinct academic health care settings, to address faculty members’ ongoing needs for community and, ultimately, to serve as a source of support for the application of new knowledge to routine education activities. A research project was launched jointly to describe the process and progress of attempting to develop communities of practice at the three sites and to identify common and unique influences on sites’ progress.

**Data Collection:** Phone interviews were conducted with group facilitators from each site following group meetings, for the duration of the initiative.

**Analysis:** Multiple case study methodology was employed to describe and compare the processes and progress of attempting to initiate communities of practice at the three sites and to identify obstacles related to organizational context.

**Findings:** All three sites made limited progress in developing a shared domain of interest and a shared history of regular interaction (i.e. regular meetings). Participants identified different professional backgrounds and different education practices as challenges to establishing shared interest. More prominently, they identified busy schedules, geographic barriers, and absence of protected time as obstacles to regular and consistent meetings.

**Discussion:** Difficulty establishing shared interest and shared history are considered in light of the unclear meaning of “education scholarship”, cognitive and ethical boundaries between professions, and time constraints within modern, highly complex academic healthcare settings.

**Conclusions:** While CoPs may appeal as self-sustaining, low-cost alternatives to formal programming, limited progress is possible without institutional investment and allowance commensurate with the implied scope and challenges.

## Background and Rationale

Since 2002, The Centre for Faculty Development (CFD), a partnership between the University of Toronto and St. Michael’s Hospital, has run a two-year longitudinal faculty development program called the Stepping Stones Teacher Development Program, aimed at building education and education scholarship knowledge and skills among clinical faculty. Over a period of two years, program participants are required to complete 26 hours of approved education workshops and a journal club focused on teaching and learning theory. As part of the journal club, participants are required to present on journal articles throughout the year and to complete two assignments: an annotated bibliography and a reflective paper on the application of material to education and scholarship practice. Most workshop options are centrally offered at the CFD, while journal club meetings occur in various locations throughout the academic health sciences system (Centre for Faculty Development, 2018; Richardson, Silver and Dionne, 2007).

In post-program evaluations of the first two cohorts of the Stepping Stones program, graduates identified perceived benefits of participation including the acquisition of new knowledge, increased confidence in, and enthusiasm for, teaching and the development of a network of educators. However, a number of graduates also reported significant difficulties applying their learning to their daily education and scholarship work and felt isolated from the broader education and scholarship community they had enjoyed during the program. These reports suggested a gap within existing faculty development programming, specifically that faculty members’ longer-term learning needs were not being addressed ( Kwan et al., 2015; Richardson, Silver and Dionne, 2007).

Stepping Stones participants’ reports are consistent with identified limitations of the traditional, currently still predominant, approach to faculty development programming. Most faculty development programs consist of structured, time-limited programs such as workshops, seminar series, short courses and some term-limited longitudinal programs, usually isolated from learners’ workplaces ( Leslie, Baker, Egan-Lee, Esdaile, Reeves, 2013; Steinert et al., 2006; Steinert, 2012; Steinert et al., 2016). Programs that build direct links to faculty members’ ongoing educational practices ( Steinert, 2006) or address the challenges of translating new learning to the workplace ( O’Sullivan and Irby, 2011) are rare. Moreover, programs are overwhelmingly designed for individual learners, with limited attention to team learning ( O’Sullivan and Irby, 2011; Steinert et al., 2006). Pointing to these gaps, several commentators have called for greater incorporation of the perspectives of lifelong learning, workplace learning and social learning within faculty development strategies ( Lieff, 2010; O’Sullivan and Irby, 2011; Steinert, 2010, 2012).

Among these recommendations have been calls for a shift towards a community of practice (CoP) perspective ( O’Sullivan & Irby, 2011; Steinert, 2010, 2012). “CoP” refers to a group of people who, through regular interaction, become better at a practice in which they are all engaged and are all motivated to improve ( Wenger, 1998). Sharing a common domain of interest, namely a practice they wish to improve, provides members’ primary motivation to participate and bonds them, helping them to develop a shared identity ( Li et al., 2009; Wenger, McDermott and Snyder, 2002; Wenger-Trayner, 2015). Over time they reflect together on their own experiences of engaging in the practice, define a shared conception of competency and develop a shared repertoire of resources to refine their work continuously and respond to emergent challenges. As they accumulate a shared history through regular interaction, they also develop a sense of mutual accountability and concern, which further solidifies a distinct and enduring group identity and generates further motivation to work together ( Wenger-Trayner 2015).

To date, CoPs have received relatively little attention as an object of study in faculty development research ( Leslie et al, 2013; Steinert, 2006, 2016). O’Sullivan and Irby (2011) interpret this paucity as an instance of a more general preference for linear, quasi-experimental study designs, in which attempts are made to measure the effects of discrete, time-limited programs on immediate individual satisfaction, attitude, knowledge, skill and behaviour. The frequent consequence, they argue, is findings that neither achieve the positivistic standard emulated, nor provide sufficient analysis of processes and contexts to guide future efforts. Correspondingly, in a recent systematic review, Steinert and colleagues (2016) identify a preponderance of quasi-experimental designs and call for greater use of qualitative and mixed methods that would allow analysis of processes of change and capture faculty members’ stories of “why” and “how” faculty development works. The authors also call for “a greater focus on the organization at large” rather than just individual learners and groups of learners, and investigation of the interplay between different features of the complex environments in which faculty development efforts occur. These suggestions echo those of Leslie et al. (2013) who call for future research to explore CoPs in the workplace and address how different organizational and contextual factors shape the success of faculty development programs.

## Intervention

Following review and discussion of Stepping Stones evaluations and faculty development literature, a group of clinical faculty members and education researchers (DK, SN, FF, JM, ML, LM, LN, DR) initiated a project with the intention of filling the identified gaps in local faculty development programming. The Teaching and Education Scholarship Community of Practice (TESCoP) was initiated to instigate CoPs within the local academic health sciences system. The hope was that, if they developed and endured, these CoPs would serve as an ongoing, self-sustaining resources for members to reflect on continuously and improve their teaching and education scholarship practice. Three teaching hospitals were included in the initiative, each of which would be the location of efforts to establish a new CoP. These sites were selected to support the research goal of examining how CoPs develop (or do not develop) in different settings. The three sites are highly distinct in terms of the populations served and the spectrum of clinical services:

**Site 1**: **A specialized academic hospital** focused on a specialized treatment domain and with an extensively developed research mandate

**Site 2**: **A community teaching health centre**, serving a particular catchment area in the city

**Site 3: A multi-site academic health sciences institution**, with a highly developed research mandate and highly diverse set of care services.

In June 2014, a Steering Committee (SC) was formed, composed of the clinical faculty members and education researchers who had initiated the project (DK, SN, FF, JM, ML, LM, LN, DR). The SC was formed to serve as a common point of contact for the three sites, monitor sites’ progress, conduct research and evaluation activities and disseminate findings. The SC met 13 times between June 2014 and August 2015

The SC was multi-professional in its composition, representing several clinical and non-clinical domains, including occupational therapy, education research, medicine, pharmacy and physiotherapy. One SC member from each hospital site served as “site lead”. Site leads participated as founding members of the CoPs at their own sites and helped to initiate them. As SC members, they also participated in SC meetings, providing a communication and coordination link between the SC and the sites. Two clinical faculty members were recruited from each site to plan and coordinate site meetings. These individuals were not members of the SC. Together with the site leads, the facilitators are referred to herein as the founding members. Like the SC, each site’s founding membership was multi-professional, representing a range of health professions, clinical practice leadership and education programming and research domains.

One preparatory workshop was held for all three sites in June 2014, during which facilitators, site leads and SC members worked to establish a common understanding of CoPs and to develop action plans for their respective sites. The specific vision, objectives, activities and structure for the three groups was left to each site’s discretion, with minimal explicit direction imposed by the SC at the outset and throughout the project. Each site was encouraged to act independently in terms of focus, recruitment, membership and frequency of meetings.

## Research Component

A qualitative study was conducted, running concurrently with the project. We employed a qualitative, multiple case study design, with each site serving as a distinct “case”. This approach was used to identify and explore context-specific and context-independent influences, nuanced benefits and challenges in the development of the CoPs and to gain in-depth theoretical insights into the processes involved within, between and across the sites ( Stake, 2006).

Our conception of process and progress were informed by two sensitizing concepts ( Bowen, 2006; Charmaz, 2006) identified within the CoP literature: shared domain of interest and shared history ( Li et al., 2009; Wenger, 2009, 2010; Wenger-Trayner, 2015). These can be understood as fundamental developmental traits of a CoP because they provide the primary mechanisms (i.e. motivations) for individuals to persist in pursuing joint activities. Hence, these concepts were deemed particularly important, given the initiative’s interest in generating CoPs as self-sustaining sources of ongoing mutual support.

## Research Objectives

1. To describe the process and progress of attempting to initiate CoPs within the three sites, with particular emphasis on the identification of a shared domain of interest and accumulation of shared history of regular interaction
2. To identify common and unique influences on processes and progress in developing CoPs at each site.

## Data Collection

Data collection lasted 10 months, between December 2014 and August 2015. Following each site meeting, a one-on-one semi-structured telephone interview was held with the facilitator or site lead who had led that meeting. Each interview was conducted by one of two research associates (FF, DM), using an interview guide. Research associates took detailed notes during and immediately following interviews. These notes served as the data source for analysis. Using plain, broadly accessible language, interviews covered:

• Attendance at the meetings (how many participants: overall; number of core members; number of new members)
• The groups’ principal focus (e.g. scholarly research, reflection on daily practice)
• Activities and developments during and between meetings
• Participants’ assessment of progress
• Obstacles and influences on group development and learning.

## Analysis

Starting with Site 1, the research team members (DM, DK, SN, DR) independently read the telephone interview notes, focusing on material pertinent to the research objectives and sensitizing concepts. The team members then discussed salient and pertinent findings for the site. Two team members (SN, DM) took turns taking analytic notes, recording differences and resolutions in interpretation. The same approach was then applied, in turn, for Site 2 and Site 3, with team members identifying similarities and differences between and across sites as analysis proceeded ( Stake, 2006). Further analysis involved iterative refinement of findings through focused coding ( Charmaz, 2006).

## Findings

A total of 14 interviews (Site 1 = 6 interviews; Site 2 = 5 interviews; Site 3 = 3 interviews) were conducted with 9 founding members (n=3/site). All interviews were conducted shortly after each site meeting.

### Site 1: A specialized academic hospital

The founding members of Site 1 were an education specialist and researcher (site lead), a social worker (facilitator 1) and a physician (facilitator 2). The group held 6 meetings during the project period.

#### Process and Progress

As the initiative progressed, the group initiated and pursued two principal activities. The first was a series of “Lunch ‘n’ Learn” events, intended as a venue to engage a broad audience within the institution in discussions about scholarly work in education. The second, a team research project, which became the group’s central activity, was pursued primarily as a means of engaging, and establishing a shared focus among team members.

In the first three months, the group experienced several challenges. One founding member indicated that building momentum, motivation and focus were proving difficult after a grant application for a research project was rejected. Subsequently, the founding members made an effort to regroup to attempt to address these issues, deciding to focus the group’s energies again on the team research project, specifically improving the study protocol and drafting a new grant application. Focusing on this activity, it was reasoned, would help the group regain focus, shared purpose and motivation.

Nevertheless, a gap of three months followed February’s site meeting. In the subsequent May meeting, leadership duties passed to a new facilitator, when the first had to reduce involvement. During this meeting, the group discussed the need to re-establish focus and motivation. To this end, a preliminary terms-of-reference was drafted, in part as a means of clarifying and standardizing facilitator duties.

Another large gap in meetings occurred after May. As of the next and last meeting in August (last during the project period), held via conference call, the group’s focus was still unspecified. Some members were primarily interested in research and scholarship projects, others in curriculum and training. The group also learned that some peripherally involved members, from professions not currently represented in the group, had decided they could no longer be involved.

#### Influences on Progress

Founding members of Site 1 focused primarily on scheduling constraints as the major obstacle to progress. Busy schedules were cited as the primary reason for the large gaps between meetings and the fact that peripherally-involved members ultimately stopped coming. One founding member speculated that time constraints were a particular challenge for salaried professionals (i.e. non-physicians), due to the relative inflexibility of their work schedules. Additionally, continuity was compromised by the fact that the original facilitator had to reduce participation significantly for an extended period of time to fulfill institutional obligations. Following the three-month gap after the February meeting, for example, the new facilitator noted a lack of clarity regarding the facilitator role and resultant loss of focus within the group.

Time constraints also appear to have frustrated the development of mechanisms or artefacts that might have facilitated continuity and shared focus even when regular meetings with the same core members were not always possible. Although a terms-of-reference was drafted by one member prior to the gap between the May and August meetings, as of August other members had not provided feedback and it had not progressed.

### Site 2: A community teaching health centre

The founding members of Site 2 were a physician (site lead), another clinician/clinical practice leader (facilitator 1) and a staff education specialist (facilitator 2). The group held four meetings during the project period.

#### Process and Progress

The founding members of Site 2 had already been involved in a previous informal group, with a clear focus on education scholarship and a history of education research productivity. They envisioned a CoP that would serve as an extension and expansion of this previous group, through the addition of a mandate to increase the profile and awareness of education scholarship within the institution. While they did wish to attract new members, their initial priority for the group was to maintain, nurture and build upon the collegial, focused, productive atmosphere of the preexisting collaboration.

To facilitate focus and productivity, the group made plans for a shared project to bring the Stepping Stones program to their institution, which they saw as a way to increase faculty development resources for professionals there. To advance TESCoP’s institutional visibility and education agenda, the group planned to make presentations throughout the year during various rounds and to hold “Lunch ‘n’ Learns” where clinicians from across the institution could share experiences, ideas and lessons about education.

The site’s efforts to bring Stepping Stones to the institution proved unviable for reasons outside their control. According to one founding member, this demoralized the group somewhat, since the plan had initially been their primary focus. Ultimately, the site ended up cancelling a number of meetings, which led to unexpected and significant gaps in time between meetings. Additionally, in several meetings that did go ahead, one or more founding members were absent. Limited progress was made in doing rounds presentations and holding “Lunch ‘n’ Learn” events.

The group did manage to attract new attendees to some meetings, primarily from the institution’s education staff. However, arriving at a shared focus with which both the founding members and these new, prospective members would be satisfied, proved insurmountable. As of the final interview in August (end of the formal project period), the founding members had decided to halt the TESCoP-associated activities planned at the outset and to reinitiate their preexisting, informal education scholarship group.

#### Influences on Progress

Founding members of Site 2 identified scheduling constraints as a major problem, which resulted in sporadic and infrequent meetings, inconsistent attendance by founding members and, ultimately, discontinuity and halted progress. Obligations to an institutional accreditation process, and the absence of protected time for participation in the group, were cited as possible explanations for this. Sporadic and infrequent meetings were, in turn, cited as an obstacle to group functioning and development of shared focus. One member, who rotated in as facilitator, reported repeatedly delaying important decisions about group activities due to the absence of one or more founding members.

Founding members also indicated a clear divergence in interest between themselves and new attendees (i.e. prospective or peripheral members), which frustrated attempts to develop or maintain a shared focus *while* expanding the group. As noted, the founding members had already formed a group prior to TESCoP, with a well-defined focus on introducing education scholarship programs to the institution and conducting education scholarship research. One founding member noted that most of the new, prospective members had a different motivation and focus. According to one interview participant, underlying this was a different practice. Rather than clinician-faculty, these individuals were part of the institution’s education staff. As a consequence of different practice needs and interests, these new individuals, perhaps quite reasonably, wanted a venue to obtain one-way consultation and advice on their daily education practice, instead of the mutually supportive, scholarly community the founding members had envisioned. As noted, this was cited as a reason for the ultimate decision to cease group expansion efforts.

### Site 3: A multi-site academic health sciences institution

The founding members of Site 3 were an occupational therapist (site lead), a speech and language pathologist (facilitator 1) and a physician (facilitator 2). The group held two meetings during the project period.

#### Process and Progress

Site 3 initially wanted to attract a large number of attendees to their meetings, because this seemed appropriate given the multi-site institution’s large size. Nevertheless, a desire was also expressed to build, among an expanding membership, a sense of ownership and commitment so that group duties could be shared. The founding members also stated interest in arriving at a common focus that was relevant to founding and prospective members’ education and practice needs.

The group decided early on that it would meet every other month, rather than every month, feeling that, given the busy schedules of founding and potential members, meeting more frequently would be unfeasible and a deterrent. For similar reasons, the group decided early on not to pursue a group project. It decided instead to focus on devoting meeting times to guest speakers. After encountering several challenges, the group amended this plan, opting for a less formal format, consisting of informal presentations by *ad hoc* attendees.

Between December and March, the founding members reported frustration due to lack of response from potential members following a large email recruitment effort. This development motivated the decision to cancel the planned February meeting. Founding members felt that a small turn-out would not justify the guest speakers’ time and effort. The group subsequently decided to abandon the guest speaker plans and try a less structured approach. The next meeting in May consisted of two informal presentations by local clinical faculty, both on patient education.

As of the May meeting, founding members reported a loss of momentum and a feeling that the group was far behind what they had initially hoped to achieve. Despite having presented various ideas for the group’s focus, they were still struggling to settle on something appealing to prospective members while maintaining their own enthusiasm. Attempts to recruit, and solicit greater commitment from, additional members had been largely unsuccessful. One founding member expressed a feeling that they were nowhere near ready to rotate facilitator duties to any new members, as they had hoped.

Two founding members reported after the May meeting that their interest and motivation were waning. Concerted efforts to coordinate meetings and recruit members, in one individual’s view, had yielded limited results. The facilitators made the decision to dedicate less time to the group subsequently and to moderate their expectations. No plans to terminate the group were mentioned but subsequent meetings within the project period were delayed and ultimately cancelled. Hence, the May interviews were the last data source for this site.

#### Influences on Progress

The founding members of Site 3 identified busy schedules and competing obligations as the primary obstacles to increasing membership and establishing meeting consistency. Busy schedules were attributed to factors such as heavy clinical caseloads and involvement in mandatory hospital initiatives. Continuous difficulty with finding meeting times when they themselves could all attend was cited as a major reason for sporadic and infrequent meetings.

Additional challenges, unique to this site, were identified as exacerbating factors. The “site” was in fact a very large conglomerate institution composed of several advanced academic health science centres. Founding members raised the possibility that low recruitment and reticence among new members to take an active role may have resulted from the sheer number of professional education programs and services available at each of these locations. There was simply a large amount of competition for peoples’ time and commitment. Additionally, they felt that the geographic distribution of the various centres throughout the city, presented a significant deterrent to attendance. One founding member emphasized the challenge of attending regularly, given the substantial distance between their work location and the meeting site.

This study was approved by the University of Toronto Research Ethics Board, protocol reference #30070.

## Discussion

This study’s findings suggest that, on the whole, despite concerted efforts among founding members, the three sites made little progress in developing CoPs that were likely to serve as sustainable sources of mutual support. Founding members of the three groups recognized the importance of shared interest and made significant efforts to establish clear foci that could bind their expanding groups. However, solidification of a shared domain of interest was, in all cases, an unresolved endeavor, with little evident progress during the project period. All three sites also experienced significant challenges in attempting to build a shared history of continuous interaction, plagued by meeting cancellations and incommensurable schedules. In all sites, discontinuity was a major theme in interviews and seems to have hindered relational, conceptual and practical continuity.

The sites’ difficulties in establishing a shared domain of interest may be partially attributable to the ambiguous or vague meaning of the practice domain. From the outset, TESCoP faced a significant challenge in that the domain(s) of practice, teaching and education scholarship encompass a potentially wide variety of activities. The inclusion of education scholarship, in particular, may have potentiated particular challenges in this regard.

These challenges may be embedded within the history of this practice concept. Boyer (1991) coined the original term “scholarship of teaching” specifically to encourage recognition and appreciation of a relatively wide range of activities, beyond traditionally recognized domains of formal research productivity. Accordingly, subsequent developments of this concept have tended to describe education scholarship in terms of the underlying characteristics of knowledge production within a community of peers, including *both* traditional, formal academic research and less formal pursuits such as localized education innovation work ( Van Melle et al., 2014). Shulman and Hutchings, for example ( Hutchings and Shulman, 2008; Shulman, 1998) described education scholarship as the deliberate planning, execution and evaluation of an education activity with or without the trappings of formal research. The essential element in this conception is the underlying principle of knowledge building within a community of peers, producing and sharing one’s work, such that it is “susceptible to critical review .. and amenable to productive employment in future work by members of that same community” ( Shulman, 1998, p. 14).

In the TESCoP project, the focus on this broadly-defined, inclusive domain of interest, made possible the participation of professionals from very different clinical and non-clinical professions, interested and engaged in very different education and knowledge pursuits. In particular, it held the promise of attracting both those whose experience and interests did and did not include formal education research. Focusing from the outset on this domain allowed a potentially rich and stimulating learning environment ( Wenger, 2009, 2010). However, this diversity and richness also implied a major challenge, for all groups, of collectively defining common aspects of their respective practices. Hence, when the initiative got underway, the abstract, general concept of education scholarship may have been confronted by the concrete particularity and variability of individuals’ real practices and practice interests.

In addition to inviting a diversity of interpretations of the domain of interest (education scholarship), the TESCoP initiative was intentionally professionally diverse from inception through execution. This may have presented an additional challenge, due to social and cognitive boundaries particular to members’ respective professions linked to how they understand education and scholarship. In addition to practice differences, professions are characterized by different value systems and distinct sets of conceptual, linguistic and interpretive repertoires, which set them apart from each other and can frustrate mutual communication ( Carlile, 2002; Ferlie, Fitzgerald, Wood and Hawkins, 2005; Kislov, Harvey and Walshe, 2011; Irvine, Kerridge, McPhee and Freeman, 2002). Interaction between professional communities can stimulate new ideas and approaches to practice but can also strain the limits of mutual intelligibility and relevance, presenting significant challenges to communication and shared purpose ( Mørk, Aanestad, Hanseth and Grisot, 2008; Wenger, 2009).

Given the magnitude of these challenges, the TESCoP initiative’s attempt to build multi-professional CoPs around an inclusive practice domain implied the need for continuous and extensive interaction and engagement over time in order to articulate the domain of practice across differences in professional context and education practices ( Wenger, 2009). A salient finding from this study is the degree to which the three groups struggled to establish a history of continuous interaction, through which this work could be achieved. Despite concerted effort and commitment, schedule constraints were reported as a pervasive obstacle to meeting regularly and predictably.

These findings are similar to those from a mixed-methods study by Zibrowski and colleagues on obstacles to the pursuit of education scholarship among medical faculty members participating in a formal education scholarship group ( Goldszmidt, Zibrowski and Weston, 2008; Zibrowski, Weston and Goldszmidt, 2008). These authors found that, despite interest and irrespective of background education, participants were able to devote only a negligible amount of time to education scholarship. The most commonly cited obstacle was lack of protected time, with focus group participants reporting the juggling of multiple obligations and the fragmented nature of opportunities to work as key challenges. Similarly, participants in the TESCoP initiative cited the absence of protected time and competing obligations as the source of scarcity and fragmentation in opportunities to meet.

Rather than simply highlighting the consequences of individual over-commitment, the challenge of over-burdened schedules draws attention to the manner in which professional learners are situated within the systems in which they work. In healthcare, there is increasing pressure for professionals to embody expertise and skill in a complete range of competency areas including clinical care, education and research ( Leslie et al., 2013). Continuous development in each of these areas requires commitment to the time and attention needed for corresponding activities and participation in multiple communities. Insofar as an individual participant is engaged in these multiple pursuits, their professional life unfolds over time, as a trajectory through the system, into, within, out of and between, communities - an ongoing journey through a “landscape of practice” ( Wenger, 2009, p. 12). Under such conditions, it is reasonable to expect that the various threads of an individual’s trajectory will entangle, with greater frequency as the extent and variety of commitments increases.

Since a CoP, or, any community, involves multiple individuals each of whom participates in other communities ( Wenger, 2010), the potential for scheduling conflict is compounded. Where an individual’s weekly, monthly or yearly schedule represents a unique trajectory through the system, a particular community, or community event, occurs at the nexus of several such unique trajectories. Consequently, to pursue collective activities and develop a history of its own, a CoP in these conditions is faced with the continual challenge of reconciling difficult-to-reconcile schedules. This is a challenge not to be taken lightly, as reflected in the severe difficulties the founding members in this study encountered in identifying and following through with mutually acceptable meeting times. The challenge was not just that one or more had no available time, but that the times they *did* have available seldom coincided. The effects of this issue were further exacerbated by the absence of protected time that could be used for participation, the presence of competing programming options, geographic obstacles to attendance and, arguably, the fact that the groups’ domains of interest were still being articulated.

## Conclusions

Our findings suggest that there was a tension between TESCoP’s inclusive and exploratory vision of multi-professionalism and education scholarship, on the one hand, and the systemic constraints governing how professionals work, collaborate and learn within the academic health-sciences system, on the other. Interpretation of the planning and programming implications of our analysis can go in two basic directions. One might conclude that the community of practice model, originally intended to describe fairly stable, centralized apprenticeship traditions, is misplaced in a modern work environment that is increasingly de-centralized and complex ( Engeström, 2013). Conversely, we might argue that the CoP model represents something essential in how professionals learn and develop, and that organizations should address work policies that hinder participation in them ( Fuller and Unwin, 2004). In either case, we suggest that, while CoPs may appeal as self-sustaining, low-cost alternatives to formal programming, limited progress is possible without institutional investment and allowance commensurate with the scope and complexity of each initiative’s vision.

## Notes On Contributors

Daniel Miller is a Research Associate working at the Centre for Faculty Development, University of Toronto and St. Michael’s Hospital. He has worked on a variety of health professions education studies. Dan has a background in philosophy and public health.

Debbie Kwan is Assistant Director, Educational Development at the Centre for Faculty Development, University of Toronto at St. Michael’s Hospital. She is also Preceptor Engagement Coordinator for the Leslie Dan Faculty of Pharmacy, University of Toronto. Debbie practices as a pharmacist at the University Health Network.

Stella Ng is the Director of Research and the Arrell Family Chair in Health Professions Teaching at the Centre for Faculty Development, University of Toronto and St. Michael’s Hospital, and a Scientist with the Wilson Centre and the Centre for Ambulatory Care Education.

Farah Friesen works as Education Knowledge Broker at the Centre for Faculty Development, University of Toronto and St. Michael’s Hospital, where she works to bridge research and programs to encourage effective knowledge sharing. Farah’s main research interest is in critically examining traditional academic performance indicators, encouraging alternative perspectives on metrics, and working towards a broader (re)definition of educational impact.

Mandy Lowe is the Senior Director of Clinical Education at the University Health Network, Strategic Advisor of the Centre for Interprofessional Education, University of Toronto and holds a status appointment as Assistant Professor in the Department of Occupational Science and Occupational Therapy, Faculty of Medicine, University of Toronto.

Jerry Maniate is the Vice President of Education for The Ottawa Hospital and Assistant Professor of Medicine at the University of Ottawa. He was previously a community-based clinician-educator (General Internal Medicine) and inaugural Chief of Medical Education, Research & Scholarship at St. Joseph’s Health Centre (Toronto).

Lakshmi Matmari is a physiotherapist at a large academic teaching hospital in Toronto with a Masters in Health Professions Teacher Education and a passion for Quality Improvement. Her current focus is on exploring the factors influencing curriculum development in a clinical setting and on creating a quality improvement curriculum for front-line staff.

Latika Nirula, PhD, is the Director of Simulation & Teaching Excellence at the Centre for Addiction and Mental Health. She is an Assistant Professor in the Department of Psychiatry in the Faculty of Medicine at the University of Toronto. She leads a team in evaluation, simulation, and faculty development.

Dr. Denyse Richardson, MD, FRCPC, MEd, is an Associate Professor at the University of Toronto in the Department of Medicine, The Dalla Lana School of Public Health and the School of Graduate Studies. As a Clinician Educator at The RCPSC and UofT, her multiple educational leadership roles and scholarship spans the continuum of medical education.
