# Supplementary material for: Initiating communities of practice for teaching and education scholarship in hospital settings: a multi-site case study
Source: MedEdPublish (2016). 2018 Jun 11;7:127. [Version 1] doi: 10.15694/mep.2018.0000127.1 (PMC10699370; doi:10.15694/mep.2018.0000127.1)
Supplement: Peer review history

# Reviewer report: Reviewer response for version 1

**DOI:** 10.21956/mep.19591.r29086

**Published:** 2019-03-19

**Authors:** Trevor Gibbs

**Affiliations:** 1 AMEE

This review has been migrated. The reviewer awarded 4 stars out of 5 An interesting paper that takes a philosophical approach to exploring communities of practice. I found the list of references particularly useful, and like my co-reviewer was left with more questions than answers, which are I feel reflective of the very dynamic nature of healthcare now-days , where everything and everybody are in a state of flux. I would look forward to reading more research on the subject from this very large group of authors.

Reviewer Expertise:

NA

No decision status is available

# Reviewer report: Reviewer response for version 1

**DOI:** 10.21956/mep.19591.r29087

**Published:** 2018-12-05

**Authors:** Richard Hays

**Affiliations:** 1 James Cook University

This review has been migrated. The reviewer awarded 4 stars out of 5 I found this paper interesting, yet surprising. It is tempting to think that communities of practice are self-defining, relatively naturally occurring and self-sustaining, because people within health care organisations work in teams and many play (for example) educational roles across different organisational units. Health professional educators are a good example of a group whose potential members have much in common, despite being in different professions and locations of large organisations. Hence I was surprised that there did not appear to be communities of practice. Do the authors know why this may be so? I was then surprised to read that attempts to facilitate development of communities of practice were not particularly successful. What are the barriers? I understand pressure to provide clinical rather than education services, but am not sure that finding a suitable time is sufficient reason. Is there a mis-match between the researchers idyllic definition of a community of practice and what is practical, achievable, and sort of working on the ground? Is an effective community of practice like effective mentorship - something that can develop only when interpersonal chemistry is right? Sorry to ask so many questions, but this is the kind of research that encourages question and debate.

Reviewer Expertise:

NA

No decision status is available
